# Treatment strategies of esophageal cancer with concurrent cervical node metastasis: a Dutch nationwide population-based cohort study

**DOI:** 10.1093/dote/doag040

**Published:** 2026-04-27

**Authors:** Maxime E Sanders, Sylvia van der Horst, Teus J Weijs, Jan Erik Freund, Stella Mook, Nadia Haj Mohammad, Bianca Mostert, Bas P L Wijnhoven, Grard A P Nieuwenhuijzen, Hanneke W M van Laarhoven, Mark I van Berge Henegouwen, Misha D P Luyer, Pieter C van der Sluis, Rob H A Verhoeven, Sjoerd M Lagarde, Suzanne S Gisbertz, Jelle P Ruurda, Richard van Hillegersberg

**Affiliations:** Department of Surgery, University Medical Center Utrecht, Utrecht, The Netherlands; Department of Radiation Oncology, University Medical Center Utrecht, Utrecht, The Netherlands; Department of Surgery, University Medical Center Utrecht, Utrecht, The Netherlands; Department of Surgery, University Medical Center Utrecht, Utrecht, The Netherlands; Department of Pathology, University Medical Center Utrecht, Utrecht, The Netherlands; Department of Radiation Oncology, University Medical Center Utrecht, Utrecht, The Netherlands; Department of Medical Oncology, University Medical Center Utrecht, Utrecht, The Netherlands; Department of Medical Oncology, Erasmus Medical Center, Rotterdam, South Holland, The Netherlands; Department of Surgery, Erasmus Medical Center, Rotterdam, South Holland, The Netherlands; Department of Surgery, Catharina Hospital, Eindhoven, North Brabant, The Netherlands; Department of Medical Oncology, Amsterdam University Medical Center, Amsterdam, North Holland, The Netherlands; Department of Surgery, Amsterdam University Medical Center, Amsterdam, North Holland, The Netherlands; Department of Surgery, Catharina Hospital, Eindhoven, North Brabant, The Netherlands; Department of Surgery, Erasmus Medical Center, Rotterdam, South Holland, The Netherlands; Department of Research & Development, Netherlands Comprehensive Cancer Organization (IKNL), Utrecht, The Netherlands; Department of Surgery, Erasmus Medical Center, Rotterdam, South Holland, The Netherlands; Department of Surgery, Amsterdam University Medical Center, Amsterdam, North Holland, The Netherlands; Department of Surgery, University Medical Center Utrecht, Utrecht, The Netherlands; Department of Surgery, University Medical Center Utrecht, Utrecht, The Netherlands

**Keywords:** cervical, chemoradiotherapy, esophagectomy, esophageal neoplasms, lymph nodes, lymphatic metastasis

## Abstract

Cervical lymph node metastasis in thoracic esophageal cancer occupies a conceptual border zone between locoregional and distant disease. Evidence guiding treatment selection in this population remains limited in Western cohorts. The objective was to evaluate treatment strategies and overall survival outcomes for patients with resectable esophageal cancer and concurrent cervical lymph node metastasis in the Netherlands. This population-based cohort study used the Netherlands Cancer Registry to identify patients with resectable thoracic esophageal or gastroesophageal junction cancer and concurrent cervical lymph node metastasis. Treatment strategies included definitive chemoradiotherapy, neoadjuvant therapy followed by surgery, chemotherapy with or without limited radiotherapy (≤30 Gy), palliative radiotherapy, and best supportive care. Kaplan–Meier analysis and Cox regression adjusted with inverse probability of treatment weighting (IPTW) were used to assess overall survival and treatment effects. Between 2015 and 2021, 412 eligible patients were identified. Median overall survival was 24 months for patients treated with neoadjuvant therapy followed by surgery, 18 months for definitive chemoradiotherapy, 15 months for chemotherapy, 7 months for radiotherapy alone, and 3 months for best supportive care. In multivariable analysis, neoadjuvant therapy followed by surgery was associated with longer survival compared with definitive chemoradiotherapy (HR 0.56 [0.34–0.91]). Similar estimates were observed after IPTW. Higher cN stage and poorer performance status were independently associated with worse survival. Subgroup analysis of neoadjuvant chemoradiotherapy versus chemotherapy within the surgical cohort showed no significant survival difference. In this nationwide cohort, management of thoracic esophageal cancer with concurrent cervical lymph node metastasis was highly heterogeneous. Although neoadjuvant therapy followed by surgery was associated with longer survival, interpretation is limited by baseline differences and potential residual confounding. These findings suggest that surgery may be considered within a multimodality strategy in carefully selected patients and warrant prospective evaluation to better define its role.

## INTRODUCTION

Esophageal cancer remains associated with substantial morbidity and mortality worldwide.^1^ For patients with locally advanced, resectable thoracic esophageal cancer without distant metastasis, multimodality treatment consisting of neoadjuvant chemoradiotherapy or perioperative chemotherapy followed by esophagectomy with two-field lymphadenectomy represents the current standard approach in Western practice, with reported 5-year overall survival (OS) rates approaching 45%–50% and median OS time of 49–66 months.^2–4^

A small subset of patients presents with concurrent cervical lymph node metastasis (CLNM) at diagnosis. The clinical interpretation of CLNM in thoracic esophageal cancer has evolved over time and occupies a conceptual border zone between locoregional and distant disease. Earlier TNM classifications considered cervical lymph node involvement as distant metastasis, which influenced treatment selection toward non-surgical strategies. In contrast, the 8^th^ edition of the TNM classification categorizes cervical paraesophageal lymph node stations as regional disease, aligning more closely with the Japanese Classification of Esophageal Cancer.^5–7^

This evolving classification reflects the longitudinal lymphatic drainage of the esophagus, which extends from the celiac trunk to the cervical paraesophageal region.^8–10^ Accordingly, metastatic spread may occur across abdominal, mediastinal, and cervical compartments, sometimes via skip metastases. However, despite these anatomical considerations, uncertainty remains regarding the biological behavior, disease burden, and appropriate management of patients presenting with CLNM, particularly in Western populations where adenocarcinoma (AC) prevails. This conceptual ambiguity extends beyond the locoregional–metastatic dichotomy and includes uncertainty regarding anatomical boundaries, nodal station definitions, and the extent of cervical involvement considered clinically relevant.

In the absence of standardized treatment recommendations, management strategies for patients with concurrent CLNM vary in clinical practice. The aim of this nationwide population-based study was to describe contemporary treatment patterns and associated survival outcomes in patients with resectable thoracic esophageal and gastroesophageal junction cancer presenting with CLNM in the Netherlands.

## METHODS

### Study design and data source

This population-based cohort study included patients with esophageal cancer with concurrent CLNM registered in the Netherlands Cancer Registry (NCR) between 2015 and 2021. The NCR captures all newly diagnosed malignancies in the Netherlands and includes detailed information on patient, tumor, and first-course treatment characteristics; vital status is obtained through linkage with municipal registries. According to the Central Committee on Research Involving Human Subjects, this study did not require Institutional Review Board approval in the Netherlands. Reporting followed the STROBE guidelines.^11^

### Patient selection

Inclusion was thoracic esophageal or gastroesophageal junction cancer staged as potentially resectable according to the AJCC 8th edition (cT1–4a, cN1–3, cM0) and coded in the NCR as having concurrent CLNM at diagnosis. Patients with cervical esophageal tumors, distant metastases, unclear histology, missing tumor location, endoscopic resection, or incomplete information on (chemo)radiotherapy were excluded. As the NCR does not provide detailed anatomical nodal level information (e.g. parajugular vs supraclavicular), laterality, or nodal burden for CLNM, analyses were based on the registry-coded presence of CLNM. No cases originally classified as distant metastatic disease (cM1) were reclassified as nodal disease for the purposes of this study.

### Variables

Extracted variables included age, sex, year of diagnosis, baseline WHO performance status, and comorbidity burden (Charlson Comorbidity Index). Tumor variables included histology, tumor location, clinical TNM stage, and differentiation grade. In the NCR, tumor location is recorded as upper, middle, or distal esophagus; tumors involving the gastroesophageal junction are classified within the distal esophagus category.

### Treatment categories and outcome

The management of patients was categorized into five main treatment options, based on clinical practices during the study period: definitive chemoradiotherapy (dCRT), neoadjuvant therapy followed by surgery (Neo + S), chemotherapy with or without limited radiotherapy (≤30 Gy) (CT), palliative radiotherapy (RT), and best supportive care (BSC). For subgroup analysis, surgical management was further subdivided into neoadjuvant chemoradiotherapy followed by surgery and perioperative chemotherapy followed by surgery.

OS was defined as the time interval from the date of disease detection to death from any cause or the last vital status update in the NCR. Patients still alive at the time of analysis were censored at the date of the last NCR vital status.

### Statistical analysis

Missing data were handled using multiple imputation by chained equations (MICE) under the assumption that data were missing at random. Twenty imputed datasets were generated using the *mice* package in R with 10 iterations per imputation. The imputation model included variables used in the analysis models, including patient, tumor, treatment, and survival characteristics. Baseline characteristics were compared between treatment groups using the chi-square or Fisher’s exact test, as appropriate. OS was estimated using the Kaplan–Meier method and compared using log-rank tests. Associations with OS were evaluated using Cox proportional hazards regression, reporting hazard ratios (HRs) with 95% confidence intervals (CIs); proportional hazards assumptions were assessed using Schoenfeld residuals.

To address measured confounding in the comparison between Neo + S and dCRT, inverse probability of treatment weighting (IPTW) was performed as a secondary analysis. Propensity scores were estimated using logistic regression including age, sex, performance status, tumor location, and clinical stage. Weighted Cox proportional hazards models were used to estimate associations with OS. Covariate balance after weighting was assessed using standardized mean differences (SMD). All analyses were performed in R (version 4.3.3); *P* < 0.05 was considered statistically significant.

## RESULTS

**Fig. 1:**
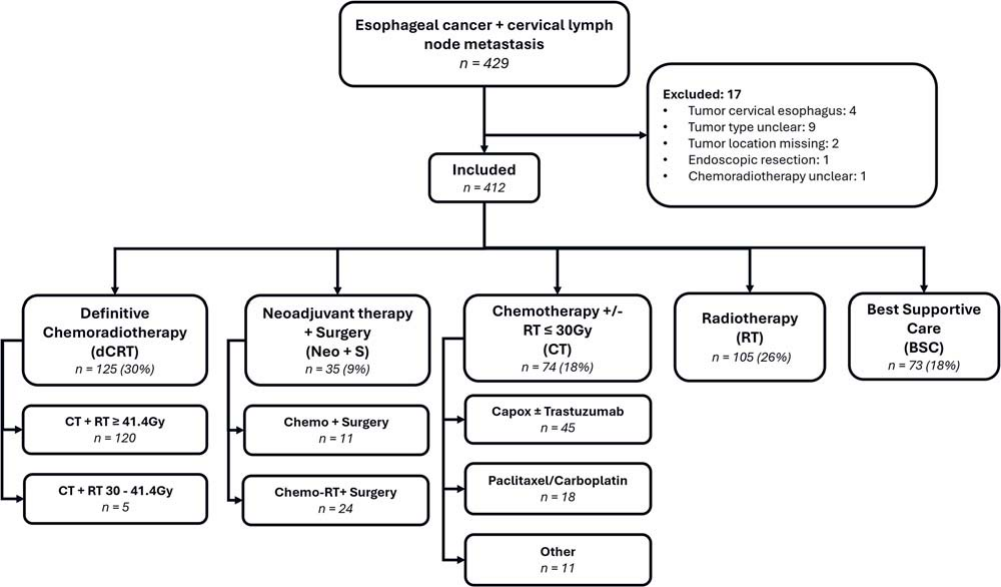
Flowchart illustrating patient assignment to treatment strategy.

From 2015 to 2021, 429 patients diagnosed with esophageal cancer with concurrent CLNM were identified from the NCR database (Fig. 1). After applying the exclusion criteria, 412 patients remained eligible for analysis (Table 1). The mean age was 69 and 67% of patients were male. AC histology was observed in 53% of cases. Most patients had cT3 disease (65%), while 40% and 42% had cN1 and cN2 disease, respectively.

**Table 1:** Patient and tumor characteristics of the included cohort

	Total	dCRT	Neo + S	CT	RT	BSC
**Total, n (%)**	412 (100)	125 (30)	35 (9)	74 (18)	105 (26)	73 (18)
**Age, year, mean (SD)**	69 ± 9	67 ± 8	64 ± 9	66 ± 8	74 ± 9	71 ± 10
**Sex**						
Male	276 (67)	85 (68)	19 (54)	57 (77)	68 (65)	47 (64)
Female	136 (33)	40 (32)	16 (46)	17 (23)	37 (35)	26 (36)
**Histology**						
AC	217 (53)	42 (33)	24 (69)	59 (80)	52 (50)	40 (55)
SCC	194 (47)	83 (66)	11 (31)	15 (20)	53 (50)	33 (45)
**Tumor location**						
Upper	61 (15)	35 (28)	3 (9)	1 (1)	14 (13)	8 (11)
Middle	121 (29)	45 (36)	11 (31)	7 (10)	33 (31)	25 (34)
Distal	230 (56)	45 (36)	21 (60)	66 (89)	58 (55)	40 (55)
**Clinical T stage**						
cT1b	2 (1)	2 (2)	0 (0)	0 (0)	0 (0)	0 (0)
cT2	72 (18)	25 (20)	6 (17)	11 (15)	20 (19)	10 (14)
cT3	267 (65)	86 (69)	26 (74)	50 (68)	68 (65)	37 (51)
cT4a	18 (4)	4 (3)	2 (6)	2 (3)	2 (2)	8 (11)
Missing	53 (13)	8 (6)	1 (3)	11 (15)	15 (14)	18 (25)
**Clinical N stage**						
cN1	163 (40)	58 (46)	22 (63)	20 (27)	40 (38)	23 (32)
cN2	176 (42)	51 (41)	13 (37)	38 (51)	48 (46)	26 (36)
cN3	57 (14)	14 (13)	0 (0)	14 (19)	12 (11)	17 (23)
Missing	16 (4)	2 (2)	0 (0)	2 (3)	5 (5)	7 (10)
**Her2Neu status**						
Negative	118 (29)	31 (25)	12 (34)	43 (58)	21 (21)	11 (15)
Positive	19 (5)	3 (2)	2 (6)	9 (12)	4 (4)	1 (1)
Unknown	275 (67)	91 (73)	21 (60)	22 (30)	80 (76)	61 (74)
**Performance status**						
WHO 0	125 (30)	55 (44)	14 (40)	31 (42)	14 (13)	11 (15)
WHO 1	143 (35)	46 (37)	16 (46)	30 (41)	35 (33)	16 (22)
WHO 2	50 (12)	11 (9)	1 (3)	3 (4)	24 (23)	11 (15)
WHO 3	12 (3)	0 (0)	0 (0)	1 (1)	7 (7)	4 (6)
WHO 4	4 (1)	0 (0)	0 (0)	0 (0)	2 (2)	2 (3)
Unknown	78 (19)	13 (10)	4 (11)	9 (12)	23 (22)	29 (40)

dCRT: Definitive chemoradiotherapy, Neo + S: Neoadjuvant therapy followed by surgery, CT: Chemotherapy without or with limited radiotherapy (≤30 Gy), RT: Palliative radiotherapy, BSC: Best supportive care, AC: adenocarcinoma, SCC: squamous cell carcinoma.

Treatment strategies varied across the cohort. Definitive chemoradiotherapy (dCRT) was administered to 125 patients (30%), neoadjuvant therapy followed by surgery (Neo + S) to 35 patients (9%), chemotherapy (CT) (± ≤30 Gy radiotherapy) to 74 patients (18%), radiotherapy (RT) alone to 105 patients (26%), and best supportive care (BSC) to 73 patients (18%).

Baseline characteristics differed notably across treatment groups (Table 1). Patients in the Neo + S group were the youngest (mean age 64 ± 9 years), while those receiving RT were the oldest (mean age 74 ± 9 years). Histology distribution varied: SCC was most prevalent in the dCRT group (66%) and least in Neo + S (31%) and CT (20%). Tumor location also differed, with upper-third esophageal tumors most common in the dCRT group (28%) but rare in Neo + S (9%) and CT (1%). Performance status varied significantly: WHO 0 was most frequent in dCRT (44%) and lowest in RT (13%) and BSC (15%), while WHO 2–4 was more common in RT and BSC, reflecting poorer baseline fitness.

**Fig. 2:**
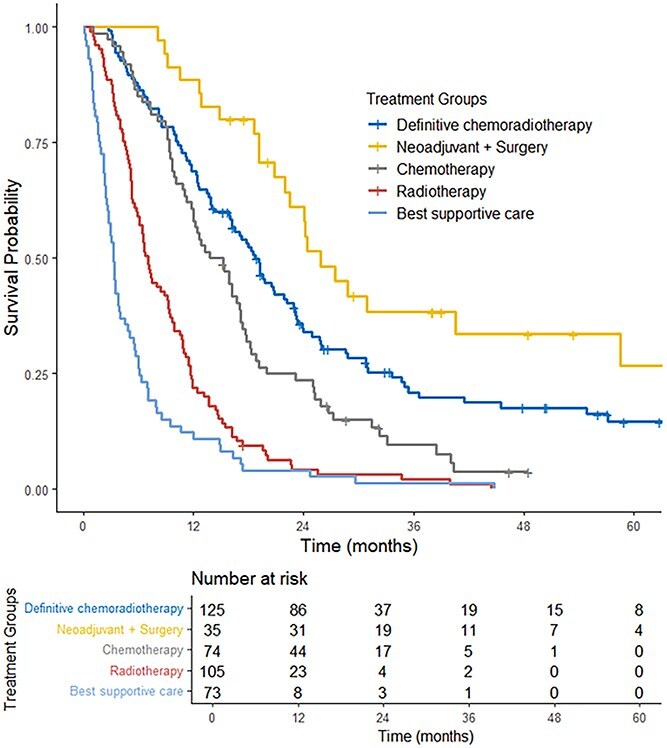
Overall survival curves for the entire cohort.

The median OS was as follows: 18.0 months for the definitive dCRT group, 24.2 months for the Neo + S group, 14.5 months for the CT group, 7.0 months for the RT group, and 3.2 months for BSC group (Fig. 2). Similarly for the groups Neo + S, dCRT, CT, RT, and BSC the 3-year OS rate was 38%, 21% 10%, 2%, and 1%, respectively. The distribution of treatment strategies across the study period (2015–2021) remained relatively stable, with no significant change in treatment allocation across years (Chi square test, *P* = 0.22) (Supplementary Fig. S1).

**Fig. 3:**
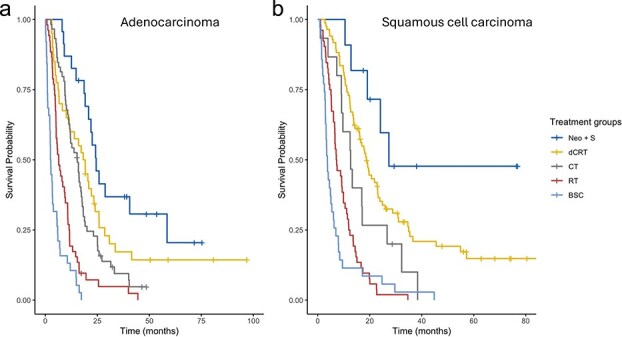
Overall survival curves for (a) adenocarcinoma and (b) squamous cell carcinoma. Neo + S: Neoadjuvant therapy followed by surgery, dCRT: Definitive chemoradiotherapy, CT: Chemotherapy without or with limited radiotherapy (≤30 Gy), RT: Palliative radiotherapy, BSC: Best supportive care.

When stratified by histology, the 3-year OS rate among patients with SCC treated with Neo + S was 46%, compared with 38% for SCC patients treated with dCRT (Table 2 and Fig. 3).

**Table 2:** Median overall survival (months) and 3-year overall survival rates

	Total		Adenocarcinoma		Squamous cell carcinoma	
	Median OS (m)	3-year OS rate (%)	Median OS (m)	3-year OS rate (%)	Median OS (m)	3-year OS rate (%)
**dCRT**	18.0	21.0	18.7	16.2	17.8	23.3
**Neo + S**	24.2	38.3	23.3	37.5	25.7	45.7
**CT**	14.5	9.6	15.3	9.5	12.6	0.1
**RT**	7.0	2.1	6.4	0.5	7.2	0
**BSC**	3.2	1.4	2.7	0	3.4	0

dCRT: Definitive chemoradiotherapy, Neo + S: Neoadjuvant therapy followed by surgery, CT: Chemotherapy without or with limited radiotherapy (≤30 Gy), RT: Palliative radiotherapy, BSC: Best supportive care, m: months.

Within the surgical group, median OS was 22.5 months for patients receiving perioperative chemotherapy followed by surgery (n = 11) and 25.1 months for those receiving neoadjuvant chemoradiotherapy followed by surgery (n = 24) (log-rank *P* = 0.59).

In multivariable Cox regression analysis, clinical N stage, performance status, and treatment strategy were independently associated with OS (Table 3). Compared with dCRT, Neo + S was associated with a hazard ratio (HR) of 0.56 (95% CI 0.34–0.91). Higher cN stage and poorer performance status were associated with increased mortality.

**Table 3:** Univariable and multivariable Cox proportional hazard regression analysis of factors associated with overall survival

	Univariable analysis			Multivariable analysis		
Variable	HR	95% CI	*P*	HR	95% CI	*P*
**cN stage**						
cN1	Ref.			Ref.		
cN2	1.20	0.96–1.51	0.114	1.20	0.95–1.52	0.123
cN3	2.10	1.52–2.89	**<0.001**	1.91	1.38–2.66	**0.001**
**Performance status**						
WHO 0	Ref.					
WHO 1	1.38	1.09–1.7	**0.006**	1.10	0.86–1.41	0.444
WHO 2	1.99	1.44–2.7	**<0.001**	1.56	1.10–2.23	**0.013**
WHO 3	4.16	2.56–6.74	**<0.001**	1.96	1.18–3.27	**0.010**
WHO 4	7.13	2.88–17.61	**<0.001**	3.40	1.34–8.6 0	**0.009**
**Treatment**						
dCRT	Ref.			Ref.		
Neo + S	0.59	0.37–0.94	**0.027**	0.56	0.34–0.91	**0.018**
CT	1.52	1.16–2.08	**0.008**	1.48	1.07–2.02	**0.016**
RT	3.28	2.46–4.37	**<0.001**	2.82	2.07–3.85	**<0.001**
BSC	5.85	4.27–8.01	**<0.001**	5.52	3.98–7.66	**<0.001**

dCRT: Definitive chemoradiotherapy, Neo + S: Neoadjuvant therapy followed by surgery, CT: Chemotherapy without or with limited radiotherapy (≤30 Gy), RT: Palliative radiotherapy, BSC: Best supportive care.

Bold values indicate statistical significance (P < 0.05).

A focused comparison between patients treated with neoadjuvant therapy followed by surgery (Neo + S) and those treated with definitive chemoradiotherapy (dCRT) demonstrated differences in OS. Median OS was 24.2 months in the Neo + S group and 18.0 months in the dCRT group, with corresponding 3-year OS rates of 38% and 21%, respectively (log-rank *P* = 0.02).

**Fig. 4:**
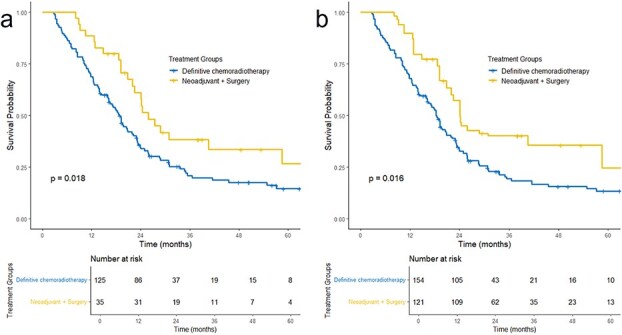
Overall survival curves comparing neoadjuvant therapy + surgery to definitive chemoradiotherapy (*P* = 0.018). (a) Unweighted and (b) using inverse probability of treatment weighting (*P* = 0.016).

A secondary analysis with IPTW was used to adjust for measured baseline differences between the Neo + S and dCRT groups. After weighting, median OS was 24.1 months for Neo + S and 18.2 months for dCRT (Fig. 4).

## DISCUSSION

This nationwide cohort demonstrates substantial heterogeneity in the management of patients with thoracic esophageal cancer and concurrent CLNM in the Netherlands. Treatment strategies ranged from best supportive care to multimodality treatment including surgery, reflecting the absence of uniform consensus and the clinical uncertainty surrounding this disease presentation.

The heterogeneity observed in clinical practice reflects the evolving conceptualization of cervical lymph node involvement in thoracic esophageal cancer, which in turn mirrors the understanding longitudinal lymphatic drainage of the esophagus.^9^ Similar to the historical reclassification of celiac lymph node metastases, which were previously designated as M1a disease before being considered regional, cervical nodal involvement remains positioned at the interface between locoregional and distant disease within staging systems.^12–15^ This persistent conceptual ambiguity continues to influence treatment selection and may partly explain the variability in management strategies and survival outcomes observed both in our cohort and across published studies.

Marked differences in OS were observed across treatment strategies. Patients treated with Neo + S had a median OS of 24 months and a 3-year OS rate of 38%, compared with 18 months and 21%, respectively, among patients treated with dCRT. In multivariable analysis, Neo + S was associated with longer survival compared with dCRT (HR 0.56), and similar estimates were observed in IPTW-adjusted analyses. These findings should therefore be interpreted cautiously and reflect associations observed in routine clinical practice rather than direct evidence of a causal treatment effect.

These findings must be interpreted in light of baseline differences between treatment groups. The association between Neo + S and longer survival may reflect, at least in part, selection of patients with more favorable disease characteristics rather than a direct treatment effect. Patients selected for Neo + S were slightly younger and had lower clinical nodal stage, with no cN3 disease observed in this cohort, whereas cN3 involvement was present in the dCRT group. Higher nodal stage and poorer performance status were independently associated with worse survival, underscoring nodal burden and patient fitness as key prognostic determinants in patients presenting with CLNM.

Histological distribution also differed between treatment groups, with AC comprising the majority of the Neo + S cohort and SCC predominating in the dCRT group. This might reflect differences in treatment selection in routine clinical practice, where histology, tumor location, and perceived disease biology may influence multidisciplinary decision-making. Consequently, patients with different histological subtypes may have been directed toward different treatment strategies, contributing to baseline differences between groups. Although histology was not independently associated with survival in the multivariable model, statistical adjustment may not fully account for the complex interplay between histology, tumor characteristics, and treatment selection. These findings highlight the difficulty of separating the effects of histology, tumor characteristics, and treatment selection in observational datasets.

The survival outcomes observed in our cohort are broadly consistent with contemporary series. In a population-based SEER analysis of patients with supraclavicular lymph node metastasis undergoing neoadjuvant therapy followed by surgery, a 3-year OS of ~39% and median OS of 25 months were reported, closely aligning with the outcomes observed in our surgical group.^16^ Interestingly this study also reported no survival difference between histology groups. Reported survival following dCRT varies across studies; median OS of 8–20 months has been described in cohorts with cervical nodal involvement, comparable to the 18 months observed in our dCRT group. ^12,16–18^

Taken together, these comparisons suggest that survival in patients with CLNM varies substantially and is influenced by a combination of treatment strategy, nodal burden, histology, and patient-related factors.

Several limitations should be acknowledged. The NCR does not provide detailed information regarding cervical nodal level, laterality, or the number of involved nodes, limiting more granular assessment of nodal burden and anatomical distribution. The relatively small number of patients undergoing Neo + S restricts adequately powered subgroup analyses. As a retrospective registry-based study, this analysis is susceptible to residual confounding and immortal time bias. Patients undergoing surgery must survive the interval between diagnosis and surgical treatment, which may artificially inflate survival estimates compared with non-surgical treatment groups. Consequently, part of the observed survival advantage associated with neoadjuvant therapy followed by surgery may reflect this methodological artifact rather than a direct treatment effect. In addition, treatment allocation in routine clinical practice is influenced by multidisciplinary decision-making, patient fitness, and disease burden, which may preferentially select patients with more favorable characteristics for surgical management. Although inverse probability of treatment weighting was applied to adjust for measured baseline differences, this approach cannot fully account for unmeasured confounding or the time-dependent nature of treatment allocation in registry-based analyses.

Beyond treatment selection, interpretation of outcomes in patients with CLNM is further complicated by variability in anatomical definitions and reporting practices. The NCR records the presence of cervical nodal involvement but does not specify nodal station, laterality, or the number of involved nodes. In clinical practice, these anatomical factors strongly influence multidisciplinary treatment decisions. As a result, patients with potentially distinct patterns of nodal disease may be grouped together within registry-based datasets, contributing to treatment selection bias and heterogeneity in reported outcomes. Furthermore, terms such as cervical, supraclavicular, and neck lymph nodes are frequently used interchangeably in the literature, often without precise delineation of nodal level or laterality. This lack of standardized anatomical reporting limits comparability across studies and may contribute to heterogeneity in reported survival outcomes. Greater consistency in defining and documenting the extent and exact location of cervical nodal involvement is essential to improve interpretability of future research.

Recognition of these limitations has prompted efforts toward more structured and detailed documentation of cervical nodal involvement within national data registries in the Netherlands, alongside prospective initiatives aimed at systematically recording nodal level, distribution, and treatment approach.^19^ Such efforts may facilitate improved risk stratification and clearer evaluation of multimodality treatment strategies in this heterogeneous patient population.

## CONCLUSION

This represents the first nationwide evaluation of treatment strategies and outcomes in patients with thoracic esophageal cancer presenting with concurrent CLNM in the Netherlands. Substantial variation in management and survival was observed, reflecting ongoing conceptual and clinical uncertainty. Although interpretation is limited by baseline differences and potential residual confounding, neoadjuvant therapy followed by surgery was associated with longer survival compared with definitive chemoradiotherapy, suggesting that surgery may represent a reasonable component of multimodality treatment in carefully selected patients. Nodal burden and patient-related factors appear central to prognosis. Standardized anatomical reporting and prospective evaluation are needed to better clarify management strategies in this complex patient population.

## Supplementary Material

doag040_Supplemental_Files

## Notes

Specific author contributions: Maxime Eduard Sanders (Conceptualization, Data curation, Formal analysis, Funding acquisition, Investigation, Methodology, Project administration, Resources, Software, Supervision, Validation, Visualization, Writing—original draft), Sylvia van der Horst (Conceptualization, Data curation, Methodology, Project administration, Resources, Writing—original draft), Teus J. Weijs (Conceptualization, Data curation, Formal analysis, Funding acquisition, Investigation, Methodology, Project administration, Supervision, Validation, Visualization, Writing—original draft), Jan Erik Freund (Investigation, Methodology, Project administration, Supervision, Validation, Writing—original draft), Stella Mook (Investigation, Methodology, Project administration, Supervision, Validation, Visualization, Writing—original draft), Nadia Haj Mohammad (Conceptualization, Funding acquisition, Investigation, Supervision), Bianca Mostert (Conceptualization, Funding acquisition, Investigation), Bas P.L. Wijnhoven (Conceptualization, Funding acquisition, Investigation), Grard A.P. Nieuwenhuijzen (Conceptualization, Funding acquisition, Investigation), Hanneke W.M. van Laarhoven (Conceptualization, Funding acquisition, Investigation), Mark van Berge Henegouwen (Conceptualization, Funding acquisition, Investigation), Misha D.P. Luyer (Conceptualization, Funding acquisition, Investigation), Pieter C. van der Sluis (Conceptualization, Funding acquisition, Investigation), Rob H.A. Verhoeven (Conceptualization, Funding acquisition, Investigation, Methodology, Project administration, Resources, Supervision), Sjoerd M. Lagarde (Conceptualization, Funding acquisition, Investigation), Suzanne S. Gisbertz (Conceptualization, Funding acquisition, Investigation), Jelle P. Ruurda (Conceptualization, Formal analysis, Funding acquisition, Investigation, Methodology, Project administration, Supervision, Validation, Visualization, Writing—original draft), and Richard van Hillegersberg (Conceptualization, Data curation, Formal analysis, Funding acquisition, Investigation, Methodology, Project administration, Resources, Supervision, Validation, Visualization, Writing—original draft)

Financial disclosure: The authors have no financial disclosures to make. There were no conflicts of interest.

Potential competing interests: None.

Funding: No specific funding was received for this study.
