# Supplementary figures and images for: Treatment strategies of esophageal cancer with concurrent cervical node metastasis: a Dutch nationwide population-based cohort study
Source: Dis Esophagus. 2026 Apr 27;39(2):doag040. doi: 10.1093/dote/doag040 (PMC13112424; doi:10.1093/dote/doag040)

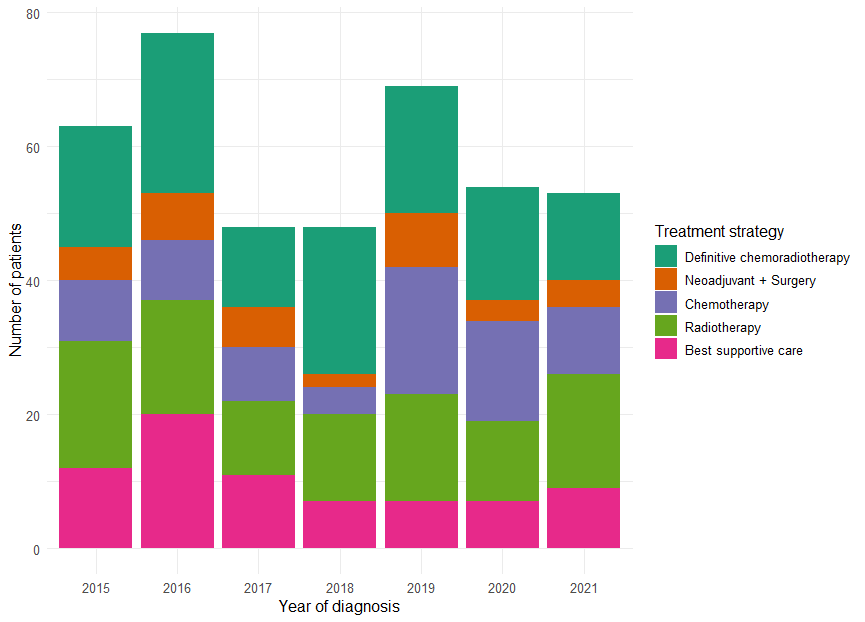


**Supplementary Figure S1.**
*Distribution of treatment strategies per year of diagnosis (2015–2021).*

Supplement: doag040_Supplemental_Files [file doag040_supplemental_files.zip › IKNL_CLNM_Nationwide_Cohort_Supplementary_File_doag040.docx]

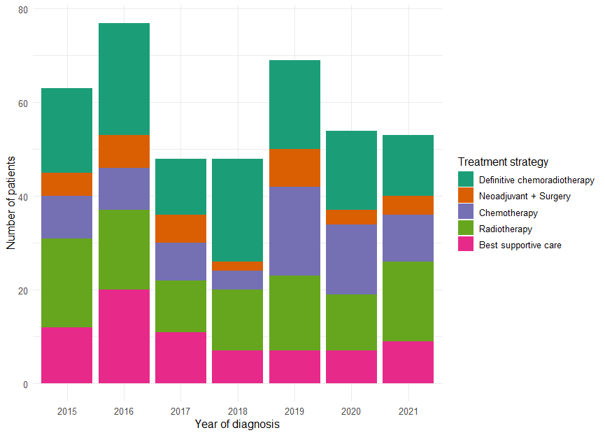

Supplement: doag040_Supplemental_Files [file doag040_supplemental_files.zip › Supplementary_Figure_S1._Distribution_of_treatment_strategies_per_year_of_diagnosis_doag040.tiff]
